# Graft versus Host Disease: From Basic Pathogenic Principles to DNA Damage Response and Cellular Senescence

**DOI:** 10.1155/2018/9451950

**Published:** 2018-03-26

**Authors:** Adam Kuba, Ludek Raida

**Affiliations:** Department of Hemato-Oncology, University Hospital and Faculty of Medicine and Dentistry, Palacky University, Olomouc, Czech Republic

## Abstract

Graft versus host disease (GVHD), a severe immunogenic complication of allogeneic hematopoietic stem cell transplantation (HSCT), represents the most frequent cause of transplant-related mortality (TRM). Despite a huge progress in HSCT techniques and posttransplant care, GVHD remains a significant obstacle in successful HSCT outcome. This review presents a complex summary of GVHD pathogenesis with focus on references considering basic biological processes such as DNA damage response and cellular senescence.

## 1. Introduction

Allogeneic hematopoietic stem cell transplantation (HSCT) offers the only curative modality for many hematological disorders. Due to advances in transplant approaches and supportive care, the use of HSCT is increasing worldwide. Despite such a progress, HSCT is still associated with substantial transplant-related mortality (TRM). Graft versus host disease (GVHD) represents the most frequent cause of TRM. GVHD occurs in about 30–50% and 70% of recipients allografted from matched related and matched unrelated donors, respectively [1].

The degree of HLA disparity between donor and recipient is a well-known and widely accepted independent risk factor for GVHD development [2]. With the growing understanding of GVHD pathogenesis, there is increasing attraction to non-HLA genotype as a tool to GVHD prediction in the last ten years [3]. Inherited genetic variants such as single-nucleotide polymorphisms (SNPs) of candidate genes, encoding various cytokines, chemokines, and inflammatory regulators, have become a subject of interest of genetic studies searching for independent predictors of GVHD development and HSCT outcome [4–7]. However, owing to the immense heterogeneity of patients' cohorts and progress in HSCT techniques in the last decade, many of reported results failed to be independently validated [6, 8, 9].

**Figure 1:**
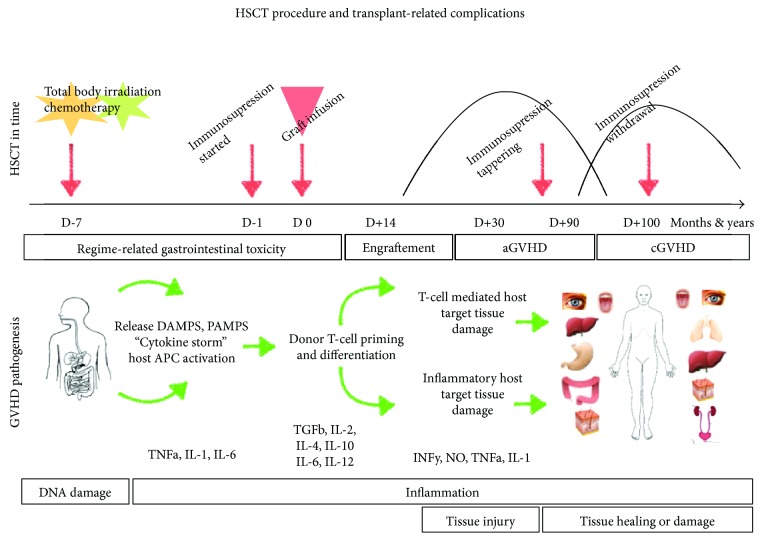
The upper part of this scheme shows transplant procedure in time with important time points of transplant management. Patients are conditioned with a variety of preparatory regimens. Shortly before graft infusion, GVHD prophylaxis (immunosuppression) is started. Gastrointestinal toxicity occurs during the neutropenic (pre-engraftment) period. Acute GVHD occurs most frequently 30–40 days after engraftment. Later occurrence is typical for late-onset aGVHD, overlap syndrome (features of aGVHD and cGVHD), or cGVHD. GVHD pathogenesis corresponding to transplant time axis is shown in 3 phase-based concepts in the middle of the scheme. Biological processes underlying GVHD pathogenesis are shown at the bottom of the scheme.

This review summarizes the updated GVHD pathogenesis linking GVHD with biological processes such as DNA damage response (DDR) and cellular senescence (Figure 1).

## 2. GVHD Pathogenesis

### 2.1. Acute GVHD

The histocompatibility differences between the donor and the recipient, the presence of donor's immunocompetent cells, and the inability of the recipient to reject these cells were defined as the basic pathogenic prerequisites for GVHD development by Billingham in 1966 [10]. Cytotoxic T lymphocytes were determined as the cellular effectors of GVHD, and the key role of antigen-presenting cells (APCs) in T-lymphocyte activation was established during the following years [11, 12]. The current understanding of aGVHD pathogenesis can be summarized as (1) initial tissue damage induced by the conditioning regimen followed by the denudation of auto- and alloantigens accompanied by massive inflammatory cytokine secretion (“cytokine storm”) activating APCs, (2) auto- and alloantigen presentation mediated by APCs together with the costimulatory signaling prime donor's cytotoxic T lymphocytes and their proliferation, and (3) the migration of activated cellular effectors toward GVHD target tissues.

#### 2.1.1. First Phase: Conditioning-Induced Tissue Damage

The conditioning-induced damage of recipients' tissues leads to danger signal secretion [13]. Besides the secretion of pro-inflammatory cytokines (TNF-alpha, IL-1beta, and IL-6), the increased expression of receptor repertoire (pattern recognition receptors, PRR) on APCs, mostly macrophages and dendritic cells, occurs as a result of the release of endogenous and exogenous antigens (damage-associated molecular patterns, DAMPs, and pathogen-associated molecular patterns, PAMPs). High-mobility group box 1 (HMGB1), adenosine-triphosphate (ATP), uric acid, heparan-sulphate proteoglycans (HSPG) as a part of extracellular matrix (ECM), and heat-shock proteins are the most significant DAMPs [13, 14]. Toll-like receptors (TLR) and other PRR expressed on APCs have the ability to sense endogenous danger signals from DAMPs and are crucial in eliciting alloreactive T-cell responses. Although the elimination of particular DAMPs diminishes aGVHD manifestation in preclinical models, such approach is controversial in clinical praxis especially in HSCT after reduced and nonmyeloablative conditionings with minimal conditioning-induced tissue damage [14].

PAMPs represent a number of pathogen-derived molecules released during the conditioning-induced disruption of natural anti-infective barriers. Lipopolysaccharides (LPS), also called endotoxins, represent the most significant ones. LPS are part of cellular membranes of gram-negative bacteria and are presented by dendritic cells (DC) and macrophages to alloreactive T cells. LPS are ligands of TLR4 playing a key role in innate immunity reactions leading to NF-*κ*B activation followed by pro-inflammatory cytokine secretion [13]. In preclinical models, the chronic exposure to LPS leads to pulmonary GVHD [15, 16]. However, interest in PAMPs mostly studied in preclinical models subsided in recent years, owing to their undetermined significance in real clinical praxis. There is much more attention being paid to the whole microbiome and its role in GVHD pathogenesis [17]. The impact of intestinal microbiome on GVHD observed in preclinical models historically has recently become a subject of detailed clinical studies due to advances in sophisticated technologies such as culture-independent rRNA gene sequencing [18]. The elimination of certain bacterial species (e.g., *Lactobacillus* spp.) and the subsequent shift of intestinal microflora in favor of pathogenic species (e.g., *Enterococcus* spp.) due to antibiotic therapy lead to dysbiosis and increased risk of GVHD development [19]. Recent studies have demonstrated that host-derived metabolic products and metabolic products of the intestinal microflora give rise to the intestinal metabolome with possible impact on pathological processes of the gut [20]. Butyrate resulting from the metabolism of complex saccharides in Clostridia directly enhances the presence of T-regulatory lymphocytes (Tregs) in the intestinal tract [21]. Tregs help maintain the intestinal homeostasis by their anti-inflammatory effect. Besides immunomodulatory properties, butyrate is a source of energy for intestinal epithelial cells and also helps maintain the recipient's intestinal barrier integrity while protecting against DAMPs and PAMPs release [22, 23]. Butyrate improves GVHD-induced intestinal epithelium damage [24].

#### 2.1.2. Second Phase: Activation of Alloreactive T Lymphocytes (Afferent Phase)

Disparities in the HLA classes I and II between the donor and the recipient play a major role in the activation of the donor's T lymphocytes. However, GVHD occurs in recipients transplanted from HLA-identical siblings as well. Thus, disparities in the minor HLA are no less important in GVHD development [25]. There have been more than 50 minor HLA identified so far [26]. The tissue specificity of minor HLA and their distribution in different tissue compartments are responsible for GVHD manifestation. Donor-derived T lymphocytes are capable of recognizing antigens on APC originating from both the donor and the recipient [27, 28]. The binding of the HLA/antigen complex to T-cell receptors (TCR) is not sufficient for T-cell activation. The final immunologic response is controlled by regulatory cosignaling between APC and T lymphocytes having either inhibitory or stimulatory effect [14]. CD28, CD40L, CD30, OX40, 4-1BB, ICOS, and LIGHT are the most significant costimulatory molecules indispensable for T-cell activation [29]. In contrary, CTLA4 or PD-1 is required for the physiologic elimination of autoreactive T lymphocytes and having inhibitory effect on T-cell activation [29, 30].

Subpopulations of T lymphocytes are equally important in the regulation of alloreactivity. Tregs and NK cells inhibit T-cell alloreactivity and diminish GVHD occurrence in preclinical as well as clinical observations [31]. T-cell activation is associated with massive cytokine secretion. Based on different cytokine profiles, CD4+ T lymphocytes are subdivided into Th1, Th2, and Th17 subpopulations. Th1 lymphocytes are involved in GVHD pathogenesis through the production of pro-inflammatory cytokines such as interferon-gamma (INF-gamma), interleukin-2 (IL-2), and tumor-necrotizing factor-alpha (TNF-alpha). However, the actual role of Th1 cytokines in GVHD pathogenesis remains unclear since Th1 cytokines exhibit variable function in different GVHD target organs [32, 33]. In contrary, Th2 lymphocytes produce IL-4, IL-11, and IL-18 that seem to be protective against GVHD development. According to latest reports, Th17 lymphocytes secreting IL-17 represent highly pro-inflammatory subpopulation capable of inducing GVHD [34]. The loss of balance between subpopulations of CD4+ T lymphocytes may influence GVHD severity [35].

#### 2.1.3. Third Phase: Chemotaxis and Target Organ Damage (Efferent Phase)

Once primed in lymphatic tissues, T lymphocytes migrate toward GVHD target tissues and organs by means of chemotaxis. Although HLA class I is expressed on all nucleated cells of the recipient, the key GVHD target organs are the GI tract, liver, and skin. There have been a lot of hypotheses concerning the site and time of GVHD onset. Organ-specific chemokines drive the migration of alloreactive T lymphocytes. Inflammatory insults elicit the expression of 4 families of chemokines (CC, CXC, C, and CX3C) at the site of GVHD target tissue. Chemokines interact with their compatible receptors expressed on lymphocytes and the recipient's tissues. The complete list of chemokines and their receptors relevant to GVHD exceeds the extent of this review [36, 37].

The final organ damage is mediated by cytotoxic cellular effectors together with inflammatory mediators. The cellular effectors possess several mechanisms of action. Interactions of CD8+ cytotoxic T lymphocytes with target cells result in the release of perforins and granzymes leading to target cell lysis. The activation of apoptotic signaling pathways Fas/FasL (CD95^−^, CD95L) and TNFR/TRAIL represents another mechanism of target cell damage. CD4+ T lymphocytes mediate their effect through Fas/FasL-induced apoptosis primarily [32]. By means of chemotaxis, neutrophils also migrate toward the site of tissue damage contributing to GVHD pathogenesis secondarily [38]. Activated macrophages colocalize with T lymphocytes at the site of tissue damage and contribute to lytic activity [38].

INF-gamma, TNF-alpha, IL-1, and nitric monoxide produced by T lymphocytes and monocyte-macrophage system are key inflammatory mediators contributing to target organ damage [32].

### 2.2. Chronic GVHD

The pathogenesis of cGVHD is much more complex, reflecting its variable clinical manifestation. Mechanisms involved in cGVHD pathogenesis partially overlap with aGVHD, especially in cGVHD developing from pre-existing aGVHD. The pathogenesis of cGVHD is based on alloreactive T-cell and deregulated B-cell interactions as well as innate immunity effectors such as macrophages, dendritic cells, and neutrophils mostly. The activation of profibrotic processes is a consequence of the aforementioned steps. The three phase-based concept of cGVHD pathogenesis is accepted currently [39].

#### 2.2.1. First Phase: Pre-Existing Inflammation

The first phase of cGVHD pathogenesis partially overlaps with aGVHD development and is mediated by innate immunity mechanisms resulting in acute inflammation and nonspecific tissue damage caused by the administration of cytotoxic medications, infections, or previous Th1- and Th17-mediated aGVHD activities. The initial tissue damage may persist, as evidenced by the progressive onset of cGVHD or overlap syndrome. Extensive tissue destruction caused by Th1 and Th17 lymphocytes leads to the release of damage molecules (e.g., ATP, nucleic acids, and HMGB1) that trigger TLR, NOD-like receptor, and inflammasome pathways [40]. The soluble form of ST2 is also released by endothelial cells, epithelial cells, and fibroblasts in response to cell damage. It works as a decoy receptor for IL-33 and drives Th2 cells to Th1-cell phenotype, which may be important in the pathogenesis of GVHD [41]. Multiple INF-inducible genes and receptors (PRRs) for PAMPS and DAMPS become upregulated at the time of cGVHD onset [42]. The INF-gamma induced expression of CXCL9, CXCL10, and CXCL11 is responsible for the recruitment of Th1 and NK cells into tissues [43]. Vascular endothelial cells (ECs) are the primary barrier separating donor and recipient tissues. ECs are the first host-derived cells to be exposed to donor immune system. If ECs express and present cognate antigens to alloreactive donor T cells, they can become susceptible to direct immune attack. Angiogenesis is critical to maintain tissue homeostasis and is modulated by multiple angiocrine factors and cytokines, which recruit inflammatory and immune cells [44]. The immunostimulatory cytosine-phosphate-guanosine (CpG) motifs in bacterial DNA bind to PRR (TLR9) resulting in B- and NK-cell activations [45]. CpG oligodeoxynucleotides (ODNs) are TLR9 agonists that show immunostimulatory effect but suppressive impact on angiogenesis [46]. CpG ODN-induced attenuation of angiogenesis is TLR9 dependent. Of interest, increased numbers of TLR9-expressing B cells associated with extensive cGVHD show hypersensitivity to bacteria-derived CpG in HSCT recipients. Also CpG response may be useful as a biomarker for both the diagnosis and evaluation of response in cGVHD treatment [47]. Apoptotic EC release LG3, a bioactive fragment of perlecan of functional importance promoting obliterative vascular remodeling [48]. Antiperlecan antibodies (anti-LG3) are accelerators of immune-mediated vascular injury [49]. Anti-LG3, endothelin-1, aminopeptidase N (sCD13), and IL-2R-alpha are biomarkers of cGVHD [50]. Importantly, anti-LG3 and endothelin-1 are considered markers of vascular inflammation suggesting that these mechanism may contribute to the pathogenesis of cGVHD, where the perturbation of microvasculature occurs [51].

#### 2.2.2. Second Phase: Deregulation of Adaptive Immunity

Thymus damage plays a key role in the second phase, manifesting as chronic inflammation and adaptive immunity deregulation. Thymus dysfunction results in decreased heterogeneity of tissue specific auto-antigens mostly present in cGVHD target organs such as the skin, liver, salivary glands, lungs, eyes, and GI tract. Consequently, donor-derived T lymphocytes possessing cGVHD antigen specificity and/or cross-reactivity expand [52]. CD4+ Tregs play a key role in peripheral and central tolerance maintenances. Tregs reconstitution is essential for the posttransplant recovery of the immune system [53]. The deficit of Tregs is associated with the significant clinical manifestation of GVHD [54].

Also B lymphocytes have a strong impact on cGVHD pathogenesis. The fate and survival of B lymphocytes is maintained by the activity of B-cell receptor (BCR) and B-cell activating factor (BAFF) [55]. Posttransplant high BAFF levels and the failure of controlling mechanisms of B-cell activation are associated with persistence and propagation of donor B lymphocytes capable of producing many auto- and/or alloantibodies [39]. Probably due to high levels of BAFF in the plasma of cGVHD patients, donor-derived polyreactive B lymphocytes are capable of escape from peripheral elimination [56]. Thus, BAFF excess expands autoreactive B cells and directly promotes TLR7 and TLR9 expressions responsible for the recognition of RNA-associated antigens and endogenous double-stranded DNA antigens, respectively [57]. Furthermore, TLR7/TLR9 signaling promotes BAFF receptor expression, thus providing a positive feedback loop [58]. There is functional synergy between BCR and TLR7/TLR9 signaling pathways, both increasing B-cell proliferation, cytokine, and autoantibody production [57]. Src kinases including Syk and Lyn kinases are proximal components of BCR signaling pathway and mediate a cross-talk between BCR-TLR pathways upon the ligation of nucleic acids containing immune complexes. Also increased BCR responsiveness with augmented Syk phosphorylation is frequently observed in B cells from patients with cGVHD compared with B cells from patients without cGVHD. The inhibition of Syk abrogates increased BCR responsiveness and CpG responses in B cells from patients with cGVHD suggesting possible novel therapeutic targets in cGVHD treatment [59]. Autoreactive antibodies produced by donor B lymphocytes are mainly targeted at minor HLA [60, 61]. Antibodies directed at antigens derived from chromosome Y (anti-HY) are often detected in male recipients with cGVHD allografted from female donors [60]. Antibodies targeted at platelet-derived growth factor receptor (anti-PDGFR) activate the generation of reactive oxygen species (ROS) inducing gene expression for collagen I followed by fibrosis in cGVHD target organs [62]. High levels of anti-PDGFR are observed in the plasma of patients with extensive cGVHD [63]. Besides antibody production, B lymphocytes possess the ability of antigen presentation or secretion of regulatory cytokines and chemokines. The actual role of B lymphocytes in cGVHD pathogenesis is more complex [62]. Recently, the identified subpopulation of B lymphocytes are CD^19+^CD^21−/+^ B-regulatory lymphocytes (Bregs), involved in cGVHD pathogenesis [64, 65]. The Bregs counts after day +100 correlate with the probability of cGVHD development [66]. Patients with active cGVHD and severe infections show significantly increased levels of immature/transitional CD^19+^/CD^21−^ B lymphocytes and significantly lower counts of memory CD^19+^/CD^27+^ B lymphocytes [67].

Regulatory natural killer (NKregs) cells are a subpopulation of NK cells with immunosuppressive characteristics. NKregs express CD^27+^ CD^11b^ low c-Kit^+^ NKp^46+^ phenotype and produce molecules with immunosuppressive functions (e.g., CTLA4, LAG-3, and PD-1). Kit^+^ NKregs indirectly reduce local antigen-presenting capacity by targeting and killing immature dendritic cells [68]. Lower proportions of CD56 bright NKregs were detected in patients with higher cGVHD frequency after filgrastim-stimulated peripheral blood apheresis and bone marrow collection, suggesting their important regulatory role in cGVHD development [69].

#### 2.2.3. Third Phase: Excessive Fibrosis

The third phase of cGVHD pathogenesis is based on deregulated processes in response to chronic inflammation resulting in excessive fibrosis, disruption of the architecture of target tissues and organs, and their dysfunction [70, 71]. Physiologic regulatory mechanisms associated with inflammatory response work to suppress and minimize cellular damage restore tissue integrity and homeostasis in order to maintain functional healing. Exuberant or excessive repair lead to fibrosis, scaring, and organ dysfunction. ECM deposition is essential for the initiation and development of healing processes. ECM represents an active factor in cell-ECM interactions. The vascular remodeling and restoration of the epithelia are the prerequisites of functional healing [72, 73]. The differentiation of fibroblasts into ECM-producing myofibroblasts is regulated by the synergism of both, innate and adaptive, immunity reactions. ECM-producing fibroblasts are activated by innate immunity cellular effectors such as myeloid cells producing TNF-alpha, IL-6, and IL-1-beta or macrophages producing TGF-beta, PDGF, or matrix-metalloproteinases (MMPs) [70]. MMPs exhibit proteolytic activities resulting in degradations of several ECM components and are important factors in tissue remodeling [74]. MMP3 is known to promote epithelial-mesenchymal transition resulting in tissue fibrosis [75]. Importantly, MMP3 plasma concentrations increase with time from cGVHD onset and are suggested as a possible biomarker of tissue fibrosis in patients with cGVHD [43]. Macrophages undergo reprogramming during the resolution of inflammation and start producing wound-healing, immune-regulatory, and angiogenic cytokines and growth factors, such as IL-10 or vascular endothelial growth factor (VEGF) [76–78]. Adaptive immunity cellular effectors Th2 and Th17 CD4+ lymphocytes activate profibrotic processes through the production of specific cytokines IL-13 and IL-17. In the preclinical setting, the contribution of B cells to cGVHD-related fibrogenesis has been well documented [79–81].

## 3. Focusing on GVHD Pathogenesis from Perspectives of Cellular Senescence

Alkylating agents and ionizing radiation used in the HSCT conditioning cause severe DNA damage. DDR signaling pathways are sensed and orchestrated by ATM and ATR kinases regulating downstream processes such as DNA repair, cell cycle arrest, cellular senescence, and apoptosis. ATM plays a crucial role in identifying DNA lesions. Rodier et al. showed that senescent cells with continuing low-threshold DDR signaling due to irreparable DNA lesions secrete a plethora of inflammatory cytokines, chemokines, growth factors, proteases, and ECM components generally known as senescence-associated secretory phenotype (SASP) [82].

### 3.1. Senescence-Associated Secretory Phenotype

ATM activation in response to DNA damage induces under certain conditions expression of various pro-inflammatory cytokines such as IL-6 and IL-8 [83]. IL-6, a cytokine with pleiotropic effects, is the most prominent SASP member [84]. IL-6 secretion is known to be associated with DNA damage-induced and oncogenic stress-induced senescence of mouse and human keratinocytes, melanocytes, monocytes, fibroblasts, and epithelial cells [85–88]. Both IL-1alpha and IL-1beta signaling pathway are upregulated in senescent endothelial cells, fibroblasts, and chemotherapy-induced senescent epithelial cells [89–92]. Additional inflammatory cytokines such as the colony-stimulating factors (GM-CSF and G-CSF) are secreted at high levels by senescent fibroblasts [86].

Extracellular soluble factors such as the MMP family are equally important subsets of SASP. Of interest, MMPs can cleave some of the monocyte chemoattractant proteins (MCP), IL-8, and a variety of CXCL/CCL family members [93, 94]. MMPs participate in the resolution of extracellular matrix fibrotic scars, being of immense importance for wound healing and tissue repair or regeneration [86, 95].

Eventually, SASP produces several insoluble ECM components such as fibronectin–a large glycoprotein found in connective tissues, on cell surfaces, and in body fluids. It interacts with other ECM components. Cells undergoing senescence in vivo display increased fibronectin expression [96].

Senescent cells can alter their microenvironment through the secretion of nonprotein substances such as ROS or nitric oxide (NO). These reactive molecules are known to reinforce senescence phenotype and to propagate DNA damage to neighbouring cells [97].

### 3.2. SASP Regulation, Expansion, and Immune System Activation

**Figure 2:**
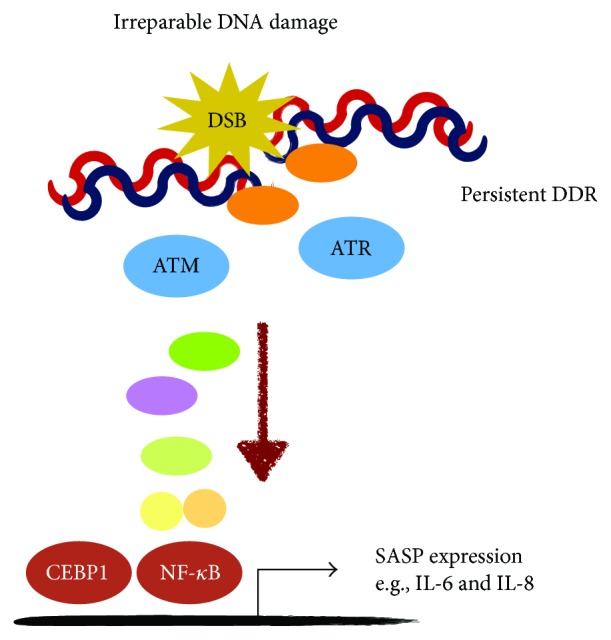
ATM kinase has a crucial role in the initiation of SASP in the DNA damage response and is required for the secretion of the two major inflammatory cytokines, for example, IL-6 and IL-8. Persistent DNA damage stimulates NF-*κ*B signaling, which consequently regulates the expression of various SASP-related genes.

Various analyses have proven that SASP gene expression is predominantly controlled by the NF-*κ*B system [98–100] (Figure 2). The NF-*κ*B activity is regulated via positive and negative feedback loops and mediated, besides other factors, by IL-1alpha and micro-RNA-146a, respectively [83]. Senescence is reinforced via positive cytokine feedback loops (IL-6 and IL-8), which help maintain the senescent phenotype.

**Figure 3:**
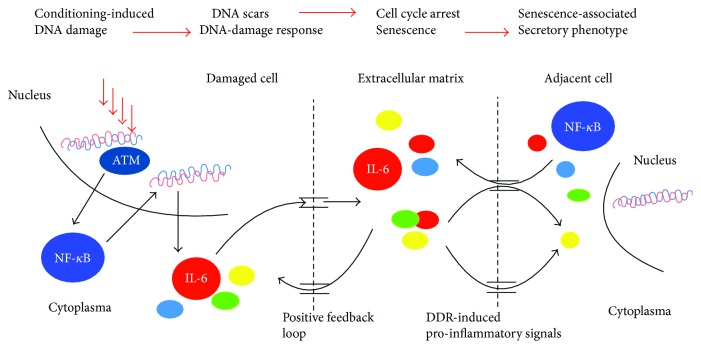
Besides other factors, cytokines released in response to DNA damage give rise to the senescence-associated secretory phenotype (SASP). These cytokines reinforce the senescent phenotype via positive feedback loops (IL-6 and IL-8), which help maintain the senescent phenotype. Also, SASP causes the surrounding undamaged cycling cells to irreversibly arrest cycling and become senescent, a phenomenon called by-stander senescence.

Also SASP causes the surrounding undamaged cycling cells to irreversibly arrest cycling and become senescent, a phenomenon called by-stander senescence [101] (Figure 3). Thus, senescent cells communicate with and modulate their microenvironment through SASP signaling. SASP components such as IL-6, IL-8, and MMPs promote tissue repair. Some SASP proteins, together with cell surface ligands and adhesion molecules expressed by senescent cells, eventually attract immune cells that kill and clear senescent cells [102]. NK cells, macrophages, and T cells participate in the clearance of senescent cells [103]. Cells that become senescent after genomic damage are known to express membrane-bound ligands for the major NK-cell receptor (NKG2D) [104].

From longer-time perspective, despite dampening the senescent activity through SASP negative regulatory feedback loops and immune clearance, senescent cells outpace the immune system and accumulate with time, producing SASP-mediated low-level chronic inflammation with both beneficial (tissue repair) and deleterious (organ dysfunction) effects [102].

### 3.3. Cellular Senescence and GVHD: Preliminary Evidence

A recently published report has documented SNPs of the *ATM* gene in association with increased risk for gastrointestinal (GI) toxicity in allografted patients [105]. ATM-rs189037 situated in the promoter region of *ATM* gene has been shown to predispose to high-grade GI toxicity in our study [105]. Accordingly, a hypothesis of defective DDR mechanisms in patients carrying predisposing variants of the *ATM* gene due to insufficient ATM production resulting in higher risk of conditioning-induced tissue damage has been postulated.

As noted, NF-*κ*B is an important regulator of innate immunity responses and also a SASP controller. NF-*κ*B system has been well established in GVHD pathogenesis [14]. In GVHD preclinical models, the inhibition of the NF-*κ*B complex member c-Rel showed the amelioration of GVHD symptoms while preserving the GVT effect [106]. Another NF-*κ*B protein subunit RelB was demonstrated to be critical for host APC compartment maturation and function and required for the expansion of donor helper T-cell type 1 (Th1). The targeted inhibition of its nuclear translocation within APC was found as a promising strategy to dissociate effector and regulatory T-cell function in the setting of Th1-mediated tissue injury [107]. Importantly, we have shown the association of two SNPs of the *NFKB1* gene encoding for the DNA-binding subunit of the NF-*κ*B complex, namely, NFKB1-rs3774937 and NFKB1-rs3774959, to be associated with GVHD development [108]. Micro-RNA-146a as the negative regulator of NF-*κ*B activation has been well documented to be involved in GVHD pathogenesis recently [5, 109]. Association studies of the IL6-174 G/C SNP with GVHD support the significance of IL-6, essential SASP factor, in various steps of GVHD pathogenesis [110–112].

As mentioned previously, also the expression of membrane-bound ligands for NKG2D by senescent cells after DNA damage corresponds with GVHD-related NKG2D expression by CD^8+^ T-cells in murine models of HSCT [113].

Telomere shortening during lifespan elicits persistent low-level DDR signaling capable of inducing cellular senescence. Interestingly, a recent study has shown that pretransplant age-adjusted telomere length correlates with TRM in allografted patients [114].

Of interest, according to our very recent data, selected immunohistological markers of cellular senescence (e.g., decreased expression of Ki67 and increased expression of p16) may improve histological diagnostics of gut mucosa obtained from patients with GI GVHD symptoms and correlate with the time of their onset, TRM, and overall survival [115].

Selected components with assigned SASP and also GVHD-related effects are summarized in Table 1 [32, 37, 39, 43, 62, 84, 86, 116–123].

## 4. Discussion

**Figure 4:**
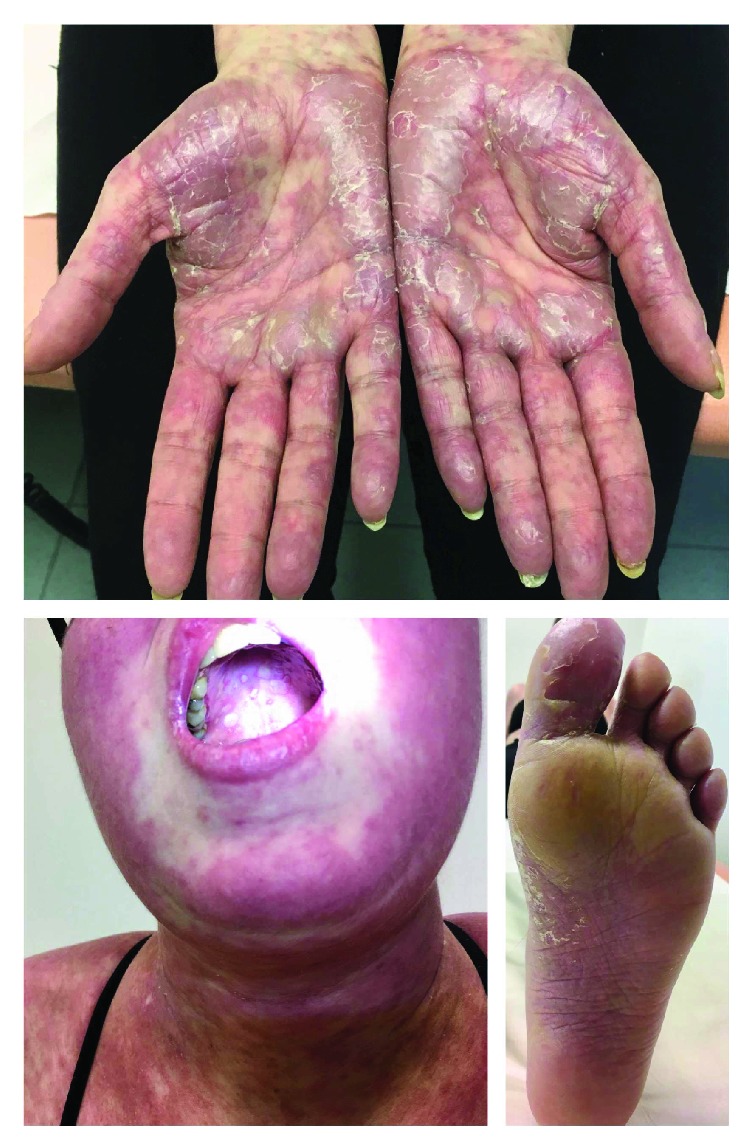
A patient presents with aGVHD after early GVHD prophylaxis withdrawal. Palmar (top) and plantar (bottom right) erythema and bullae formations are typical skin manifestations of aGVHD. The erythema of the face spreads to the neck, chest, and shoulders often resulting into generalized erythroderma. aGVHD manifestation in the oral cavity involves stomatitis and cheilitis (bottom left). The face shows signs of cushingoid features resulting from adverse side effects of corticosteroids.

**Figure 5:**
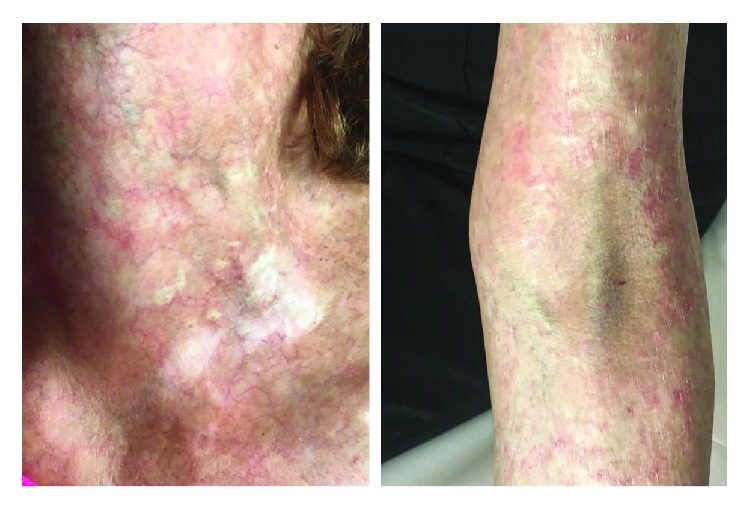
A patient after allogeneic HSCT with steroid-refractory cGVHD with dominant skin involvement (poikilodermic and sclerodermic lesions) of the neck (left) and the cubital region (right).

Despite advances in transplant techniques and posttransplant care, GVHD remains the most challenging obstacle in the whole process of allogeneic HSCT. Understanding GVHD pathogenesis has dramatically evolved during the last 50 years. Nevertheless, GVHD diagnostics are still mostly based on the careful examination of general and often nonspecific clinical signs and symptoms (Figures 4 and 5).

The lack of specific biomarkers makes GVHD differential diagnostics difficult and may lead to misdiagnoses and less than 50% response rate to the first-line treatment [124]. Novel insights into GVHD pathogenesis have not come up with new predictors of GVHD refractoriness. Clinical observations conclude that patients with advanced aGVHD are at highest risk for steroid refractoriness [125]. The mortality of patients with clinically severe aGVHD reaches 90% [126]. Only 20–30% of patients with refractory GVHD survive one year [124]. The further escalation of immunosuppression is rather deleterious and is associated with poor HSCT outcome, due to infectious complications and the suppression of GVT effect resulting in increased relapse/progression rate [127].

The GI tract harbors a substantial part of the immune system and is a frequent site of aGVHD manifestation. However, there are many other inflammatory processes including opportunistic viral reactivations in severely immunosuppressed patients [128, 129]. Lerner's histopathological classification and its modifications are generally used for GI GVHD [130–132]. However, strong interobserver variability exists [133].

Cellular senescence refers to essentially irreversible cell cycle arrest in response to oncogenic stress, a mechanism formally described as limited growth of human cells in culture by Hayflick more than 50 years ago [134]. Since then, the perception of the mechanisms of cellular senescence has evolved. According to the theory of antagonistic pleiotropy: a biological process that was selected to promote fitness in younger organisms can be deleterious in elder organisms [135]. Likewise, cellular senescence is known to promote tumor suppression and wound healing in young organisms but becomes detrimental with age, most likely by promoting chronic inflammation [116].

The hypotheses mentioned above and supported by so far limited clinical evidence provide suggestions that cellular senescence—a phenomenon in the biology of aging—may contribute to GVHD pathogenesis. These processes may also elucidate mechanisms regulating the time and character of GVHD onset as well as prediction of its therapeutic responsiveness.

## Figures and Tables

**Table 1:** Summary of selected factors involved in SASP production and GVHD pathogenesis.

Factor	Symbol	SASP-related activity	GVHD-related activity	References
Interleukin-6	IL-6	Inflammation, autocrine growth arrest, cell migration/invasion	Initial cytokine storm	Reviewed in Campisi [116] and Paczesny et al. [32]
Interleukin-8	IL-8, CXCL8	Inflammation, autocrine growth arrest, cell migration/invasion	Increased in cGVHD	Reviewed in Campisi [116] and Pidala et al. [119]
Interleukin-1	IL-1	Positive feedback component, positive regulator of NF-kB, IL-6 and IL-8	Initial cytokine storm, secreted by macrophages during the inflammatory effector phase of aGVHD	Reviewed in Coppé et al. [84] and Paczesny et al. [32]
Monocyte chemoattractant proteins (CCL chemokines)	MCPs, CCLs	Inflammation, autocrine and paracrine growth arrest, cell migration/invasion	Expressed on GVHD target organs	Coppé et al. [86], reviewed in Castor et al. [37]
Eotaxin-3	CCL26	Chemokine upregulated in senescent cells	T-cell activation marker	Coppé et al. [86], Luft et al. 2011
Matrix metalloproteinase(s)	MMPs	Tissue remodeling, wound healing, resolution of fibrosis, cell migration/invasion	MMP-3, cGVHD biomarker	Reviewed in Campisi [116], Yu et al. [43]
Fibronectin		Interacts with ECM molecules and affects cell adhesion and survival growth and migration	Chronic cutaneous GVHD	Reviewed in Coppé et al. [84], van der Straaten et al. [121]
Collagens	Col	ECM, fibrosis	Collagen deposition in cGVHD including bronchiolitis obliterans	Reviewed in Coppé et al. [84] and Cooke et al. [39]
Amphiregulin	AREG	Cell proliferation	Increased in late aGVHD	Reviewed in Campisi [116], Holtan et al. [117]
Vascular endothelial growth factor	VEGF	Angiogenesis, endothelial cell migration and invasion	Decreased in patients with steroid-refractory GVHD	Reviewed in Coppé et al. [84], Holtan and Arora [117]
Keratinocyte growth factor	KGF (FGF7)	Stimulation of cell migration and invasion	T-cell homeostasis, immune recovery, thymic regeneration	Coppé et al. [86], Chaudry et al. 2016
Epidermal growth factor	EGF	Angiogenesis, stimulation of cell migration and invasion	Decreased in patients with steroid-refractory GVHD	Tonini et al. 2003, Holtan et al. [118]
Placental growth factor	PIGF	Angiogenesis	Increased in patients with steroid-refractory GVHD	Coppé et al. [86], Holtan et al. [118]
Nitric oxide	NO	Modulator of cellular phenotype, differentiation of monocytes, promotes DNA damage and aging	Secreted by macrophages during the inflammatory effector phase of aGVHD	Rewieved in Coppé et al. [84] and Paczesny et al. [32]
Reactive oxygen species	ROS	Modulators of cellular phenotype, differentiation of monocytes, promote DNA damage and aging	Autoantibodies associated with cGVHD induce ROS accumulation and induce Col-1 expression	Reviewed in Coppé et al. [84] and Socié et al. 2017
